# Unique and Under Pressure: Conservation Genetics of an Isolated Alpine Salamander Population

**DOI:** 10.3390/biology14101428

**Published:** 2025-10-17

**Authors:** Stephan Koblmüller, Sylvia Schäffer, Raphael Donabaum, Irmgard Sedlmayr, Werner Kammel, Eva Bernhart, Lukas Zangl

**Affiliations:** 1 Institute of Biology, University of Graz, Universitätsplatz 2, 8010 Graz, Austria; sylvia.schaeffer@uni-graz.at (S.S.); raphael.donabaum@uni-graz.at (R.D.); irmi.sedlmayr@gmx.at (I.S.); lukas.zangl@gmx.at (L.Z.); 2 Technisches Büro für Biologie, Im Erlengrund 6, 8410 Wildon, Austria; office@wernerkammel.at (W.K.); eva.bernhart@medunigraz.at (E.B.)

**Keywords:** amphibia, Koralpe, microsatellites, mtDNA, phylogeography, population genetics, *Salamandra atra*

## Abstract

**Simple Summary:**

The Alpine salamander (*Salamandra atra*) is a mountain-dwelling species with limited dispersal and a preference for cool, moist habitats. A recently discovered population in the Koralpe mountain range (southeastern Austria) is geographically isolated from other known populations. Genetic analyses revealed that these salamanders are both distinct and highly structured, even across short distances, indicating limited gene flow. This isolation may increase their vulnerability to habitat disturbance and climate change. However, the population also carries rare genetic variants, making it important for the species’ overall genetic diversity. Peripheral and isolated populations like this can enhance a species’ evolutionary potential and adaptive capacity. Therefore, conserving the Koralpe population is essential—not only for local biodiversity, but also for maintaining the long-term resilience of the species.

**Abstract:**

The Alpine salamander (*Salamandra atra*) is a cold-adapted amphibian with low dispersal capacity, endemic to the Alps and Dinarides. Isolated populations at the range’s margins are especially vulnerable to habitat fragmentation and genetic erosion. We investigated the population genetic structure of *S. atra* in the Koralpe, a biogeographically important mountain range in southeastern Austria. Using mitochondrial DNA, we found that the Koralpe population harbors unique genetic variants not shared with other known populations. Strong genetic differentiation and low connectivity among Koralpe subpopulations, inferred from microsatellites, indicate long-term isolation, likely caused by unsuitable intervening habitats and the species’ limited dispersal capacity. Although the estimated effective population size (Ne = 245) is moderate and no severe genetic bottlenecks were detected, subpopulation sizes are likely small. These findings highlight the conservation value of this peripheral population and support its recognition as a distinct management unit. In situ protection, improved landscape connectivity, and continued (genetic) monitoring are essential for the population’s long-term survival. Given its unique genetic signature and pronounced structuring, targeted conservation measures are critical—especially under increasing pressure from climate change and habitat degradation. Preserving this isolated lineage will contribute to local biodiversity and help safeguard the evolutionary potential of *S. atra* as a whole.

## 1. Introduction

Biodiversity loss is one of the most pressing global challenges, with consequences that reach far beyond species extinction [1,2,3]. It often begins with reductions in population size, biomass, and genetic diversity [4,5], long before declines in species numbers become apparent. Genetic diversity, a core component of biodiversity [6], allows species and populations to adapt to potential environmental changes, cope with various stressors, and avoid inbreeding problems [7]. Maintaining this diversity requires sufficiently large, connected populations within intact habitats [8]. As emphasized by the EU Biodiversity Strategy 2030, halting biodiversity loss and achieving favorable conservation status for species and habitats—including the preservation of genetic diversity—requires reliable data on species distribution, population size, and connectivity [9]. However, such data remain scarce for many taxa, even in comparatively well-studied regions like Central Europe [10,11]. Genetic monitoring therefore plays a vital role in identifying early warning signs of population fragmentation, genetic erosion, and reduced gene flow, which can precede observable declines in population size and range.

**Figure 1:**
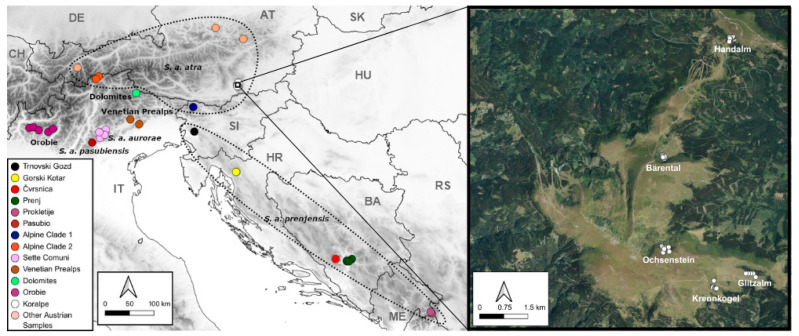
Origin of *Salamandra atra* samples included in the present study, with a lineage assignment according to [14], and with a detailed picture of the habitat and sampling sites at the Koralpe, Austria, the main study region. Countries are indicated by their two-letter codes: AT, Austria; BA, Bosnia and Herzegovina; CH, Switzerland; DE, Germany; HR, Croatia; HU, Hungary; IT, Italy; ME, Montenegro; RS, Serbia; SI, Slovenia; SK, Slovakia.

The Alpine salamander (*Salamandra atra*) is a rather secretive, entirely terrestrial viviparous amphibian. The global occurrence of the species is limited to the mountain ranges of the Alps and the Dinarides [12], where it inhabits meadows and woodlands at altitudes between 600 m and 2400 m, with known extremes of 430 m and 2800 m [13]. Austria is located in the center of the distribution area and accounts for almost half of the total distribution range [12], making Austria particularly responsible for the conservation of the species. Currently, four subspecies and three further genetic lineages are recognized within *S. atra*, several of which have restricted distribution ranges at the southern edge of the Alps (Figure 1, [14]). All Austrian *S. atra* are believed to belong to the nominal subspecies *Salamandra atra atra*. The species is listed in Annex IV of the Habitats Directive [15] and is strictly protected under the relevant conservation laws of the federal states of Austria. The species is classified as NT (Near Threatened) according to the Red List of Austria [16], as well as in the new Red Lists of the federal states Styria [17] and Carinthia [18]. Already early in this century, a significantly negative population development (33–40% area loss in Austria since 1980) was reported [16]. The Alpine salamander is a species with low dispersal capacity. Therefore, isolated populations with small population sizes and little genetic exchange with other populations are often found, especially in the peripheral areas of its distribution range [14,19,20,21]. Such a geographically isolated occurrence was recently found on the Koralpe [22], a mountain range located in the southeastern Austrian Alps. The Koralpe is a biogeographically significant region and a hotspot of endemic species [23,24]. Parts of the southeastern Alps are believed to have served as peripheral refugia during the Last Glacial Maximum, offering suitable microhabitats for the persistence of cold-adapted subalpine and alpine species [25,26,27]. This long-term stability has contributed to elevated levels of endemism in many taxa from this region. Against this background, the isolated population of *S. atra* from the Koralpe [22] provides a unique opportunity to investigate the population’s genetic consequences of historical isolation, restricted geneflow, and potential conservation concerns at the southeastern periphery of the species’ range.

In this study, we investigated the genetic structure and diversity of the isolated *S. atra* population from the Koralpe. Specifically, we used (i) mitochondrial DNA—partial mitochondrial cytochrome b and control region—to place these salamanders in a larger phylogeographic framework covering large parts of the species’ distribution range, and (ii) microsatellite markers to evaluate fine-scale population structure, connectivity, and effective population size of *S. atra* from the Koralpe. By integrating these marker types, our goal was to uncover signs of historical isolation, limited geneflow, and potential conservation concerns in *S. atra* from the Koralpe.

## 2. Materials and Methods

### 2.1. Sampling and DNA Extraction

Sampling was conducted under the permits ABT13-53S-7/1966-166 and ABT13-45661/2021-5 in areas with known occurrences of *S. atra* [22] and a potentially suitable habitat on the Koralpe mountain range, southeastern Austria, in summer 2022. Buccal swabs were collected from 94 georeferenced adult specimens caught in five main areas in the study region (Figure 1, Table S1), spanning an altitudinal range of 1652 m–1923 m, and immediately put in a DNA/RNA Shield (Zymo Research, Irvine, CA, USA) buffer.

Whole genomic DNA was extracted using the Quick-DNA Miniprep Plus Kit (Zymo Research, Irvine, CA, USA) following the manufacturer’s protocol for samples in DNA/RNA Shield buffer.

### 2.2. Mitochondrial DNA

To place the *S. atra* from the Koralpe in a larger phylogeographic context, partial mitochondrial cytochrome b (cytb) and control region (CR) sequences were obtained from five samples per main area in the study region. They were analyzed together with newly generated data for three additional Austrian samples from [28] and previously published sequences of *S. atra* covering large parts of the species’ distribution range [14,21,29,30] (Figure 1, Tables S1 and S2). One haplotype (H23) published by [14] was not used due to numerous ambiguities in the DNA sequence. PCR amplification (two overlapping fragments for cytb, one single fragment for CR) and chain termination sequencing followed [31], using the primer pairs Sa_Cytb_12F/Saat-H550, Saat-L129/Saat-Cytb1046R, and L-Pro-ML/H-12S1-ML for the two cytb fragments and CR, respectively [14,29]. DNA fragments were visualized on an ABI 3500 XL Genetic Analyzer (Thermo Fisher Scientific, Waltham, MA, USA) and trace files were checked, edited, and aligned in MEGA11 [32] and uploaded to GenBank (accession numbers PX021649-PX021704). As our sequences were slightly longer than the sequences used in [14,21], alignments were trimmed to match the sequence length of the shortest sequences in their datasets (cytb, 962; CR, 690 bp) and concatenated. Based on the concatenated alignment, a statistical parsimony network [33] was inferred in PopArt 1.7 [34]. In addition, a maximum likelihood (ML) tree was inferred in IQ-TREE 1.6 [35], employing the best-fitting model of evolution, as inferred using ModelFinder [36] and the ultrafast bootstrap method ([37]; 10,000 bootstrap replicates) and using *S. corsica* (GenBank acc. No. MF943388 for mitochondrial genome [38]) and *S. salamandra* (GenBank acc. No. EU880331 for mitochondrial genome [39]) as outgroups. FigTree 1.4.2 (available at http://tree.bio.ed.ac.uk/software/figtree (accessed on 26 August 2025)) was used to visualize the tree.

### 2.3. Microsatellite Markers

Nine microsatellite loci were chosen for a population genetic analysis of *S. atra* from the Koralpe: SST-C3, SST-E11, and SST-G6 [40]; and SalE6, SalE7, SalE8, SalE12, SalE14, and SalE23 [41]. These microsatellite loci are commonly used in population genetic studies of the target species and closely related taxa [20,21,42,43]. Forward primers were labeled with the fluorescent dyes FAM, ATTO550, or ATTO565. The microsatellites were amplified in single- or multiplex assays (Table S4) in reaction cocktails comprising 1 µL DNA extract, 2.5 µL Type-it Multiplex PCR Master Mix (Qiagen, Hilden, Germany), 0.5 µL primer mix (see Table S4), and 2 µL HPLC water, up to a total volume of 6 µL. For all amplifications, potential contamination was checked using negative controls. PCR success was checked on a 2% agarose gel, prior to genotyping on an ABI 3500 XL Genetic Analyzer (Thermo Fisher Scientific, Waltham, MA, USA). Allele sizes were scored in GENEMAPPER 3.7 (Applied Biosystems). PCR triplicates (in problematic samples up to three triplicates) were performed per sample/locus to ensure the detection of all alleles. A heterozygous genotype was confirmed if at least two independent PCR reactions detected both alleles, while a homozygous genotype was accepted if three independent PCR reactions consistently showed the same single allele.

We checked for the potential occurrence of null alleles and scoring errors with MICRO-CHECKER 2.2.3 [44]. Microsatellite variability within populations and across the whole study area was estimated by the number of alleles (NA), observed (H_O_) and expected (H_E_) heterozygosity using Arlequin 3.5.2.2 [45]. Arlequin was also used to test for the Hardy–Weinberg equilibrium (Markov chain of 100,000 steps following 1000 dememorization steps) and calculate linkage disequilibrium between loci (10,000 permutations). Population differentiation was estimated by F_ST_ [46] in Arlequin. Significance values for *p*-values were adjusted using the Benjamini–Hochberg false discovery rate method [47]. Population genetic structure was also analyzed using a Bayesian clustering approach implemented in STRUCTURE 2.3.3 [48], in which we applied an admixture model with correlated allele frequencies among populations [49] coupled with a LOCPRIOR model [50]. We did 10 Markov chain Monte Carlo (MCMC) runs of 1,000,000 steps after a burn-in of 100,000 steps for each K populations (K = 1–6). The number of genetic clusters was inferred by examining the mean and variance of the log-likelihood for each K [48] and the DeltaK statistic [51] in STRUCTURE HARVESTER [52]. Likelihoods of cluster memberships were averaged across all runs per K using CLUMPAK [53]. We also investigated the population genetic structure by a discriminant analysis of principal components (DAPC) using the R package adegenet 2.1.11 [54,55]. Groups were defined a priori based on sampling location. To avoid overfitting, the optimal number of principal components (PCs) used for the DAPC was determined by cross-validation. We used the lowest number of components with the greatest power of discrimination (20 PCs were used).

Recent migration between population pairs was inferred with BayesAss 3.0.5 (BA3 [56]). File conversion for input in BayesAss was performed with PGDSpider 3.0.0 [57]. The BA3MSAT analysis in BayesAss was run for 60,000,000 iterations, of which 5,000,000 were discarded as burn-in, with sampling every 1000 iterations. The analysis was repeated 10 times with different random seeds and setting the mixing parameters to -a 0.3, -f 0.6, and -m 0.8 for allele frequencies, inbreeding coefficients, and migration rates, respectively. With these values, we achieved acceptance rates for these parameters within the optimal range of 20 and 60% in MCMC chains, as recommended in the BayesAss 3 manual. Chain stationarity and parameter convergence were evaluated in Tracer 1.7 [58]. Effective sample sizes (ESS) > 200 indicated that the parameter log file accurately reflected the posterior distribution [59]. Following [60], we calculated the Bayesian deviance [61] to find the run that provided the best fit using the R script provided in [62]. The results from BayesAss were visualized using the R package Circlize [63]. General patterns of connectivity among the five sampling areas were visualized via a weighted network graph based on F_ST_ values in EDENetworks 2.18 [64]. In addition to visualizing the network with the automatic percolation threshold of 0.3, we reconstructed a network setting a manual threshold of 0.1, thus also allowing for the visualization of weaker connections among nodes.

The contemporary effective population size (N_e_) of *S. atra* from the whole Koralpe (all five sampled areas together) was estimated using the linkage disequilibrium (LD) method [65] in NeEstimator 2 [66], a method that was shown to produce the most accurate estimates across a broad range of demographic scenarios [67]. As recommended for this method, singletons (alleles present only in a single heterozygote) were excluded [68]. To test for excess heterozygosity, which would indicate a drastic recent reduction of N_e_ [69], we employed the two-phase model–TPM; 95% stepwise mutation model (SMM), 5% infinite alleles model (IAM); variance of geometric distribution for multi-steps mutations set to 12–implemented in Bottleneck 1.2 [70]. Significance was assessed via 10,000 coalescent simulations and one-tailed Wilcoxon sign rank tests [70]. In addition, we looked for mode shifts in the allele frequency distribution, which is also indicative of a recent genetic bottleneck [71].

## 3. Results

### 3.1. Mitochondrial DNA–Phylogenetic Placement of Salamandra atra from the Koralpe Mountain Range

**Figure 2:**
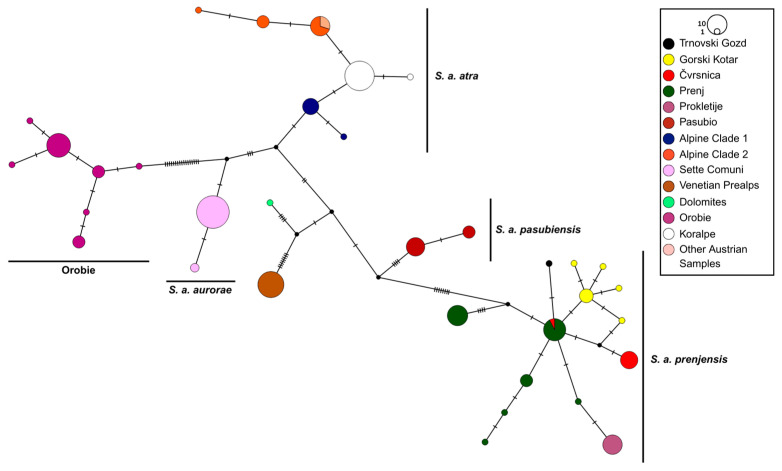
Statistical parsimony network based on concatenated cytb and CR sequences of 195 *Salamandra atra* from 35 sampling sites across the species’ distribution range. The dashes along the branches of the network refer to the number of substitutions between haplotypes. Small black dots indicate missing haplotypes.

Two mitochondrial haplotypes were found among the 25 samples from the Koralpe. One of these haplotypes was present in a single sample from Bärentalalm only. In general, salamanders from the Koralpe clustered within the subspecies *S. a. atra*, but shared no haplotypes with other Austrian, Italian, or Slovenian samples from this subspecies (Figure 2 and Figure S1). The phylogenetic relationships among the main *S. atra* lineages received only low statistical support (Figure S1).

### 3.2. Microsatellites–Population Structure of Salamandra atra in the Koralpe Mountain Range

For 90 out of 94 samples, data sufficient for the subsequent population genetic ana-lyses could be generated. Four samples had to be excluded due to insufficient amplification success. The microsatellites markers exhibited low-to-moderate levels of polymorphism, with 2–15 alleles per locus across all five populations and 1–9 alleles per locus and population (Table 1). Heterozygosities ranged from 0 to 0.923 per locus and population (Table 1), with means of 0.584 observed and 0.639 expected heterozygosity across loci and populations. A significant departure from Hardy–Weinberg expectations was detected at SalE14 in the whole dataset. As this locus did not deviate from Hardy–Weinberg expectations in any of the five sampling areas, we included it in all subsequent analyses. There was no significant linkage disequilibrium between any of the nine loci. Null alleles, as suggested by an excess of homozygotes, may be present at locus Sal27 in the Krennkogel population. As this concerned a single locus in a single population, we did not treat this locus differently in our population genetic analyses. Also, null alleles do not have a large effect on population assignment tests [72].

**Figure 3:**
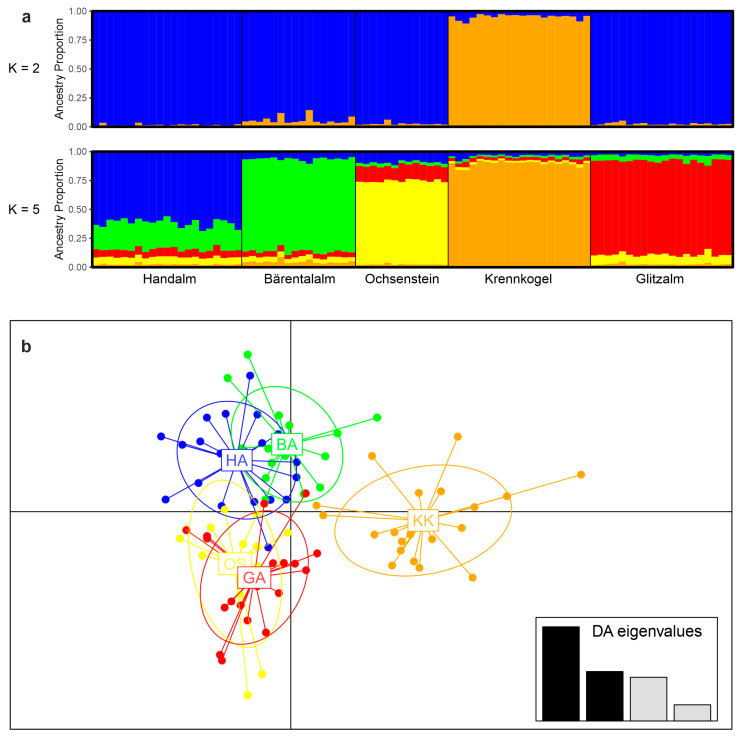
Clustering of *Salamandra atra* populations from the Koralpe, Austria, based on microsatellite data. (**a**) Bayesian cluster analysis (STRUCTURE). The two best clustering schemes (K = 2 based on the ΔK statistic [51]; K = 5 based on maximum log-likelihood [48]) are shown. Each vertical bar corresponds to one individual and the colored sections of each bar represent the individual’s assignment probability to the reconstructed K genetic cluster. Populations are separated by black lines. (**b**) Grouping by discriminant analysis of principal components (DAPC). HA, Handalm; BA, Bärentalalm; OS, Ochsenstein; KK, Krennkogel; GA, Glitzalm.

Pairwise population comparisons revealed significant F_ST_ values between all five sampling areas (Table 2), even though the sample size per population was sometimes rather small. Likewise, the Bayesian clustering approach in STRUCTURE revealed a maximum log-likelihood for K = 5 (Figure S2a), indicating the presence of five distinct genetic clusters that correspond to sampling regions. Based on the STRUCTURE output, there was no indication of ongoing geneflow among any of the five populations (i.e., individuals with mixed cluster membership; Figure 3a). Interestingly, however, the DeltaK statistic peaked at K = 2 (Figure S2b), with one cluster including the samples from Krennkogel and the other cluster including the remaining samples (Figure 3a). DAPC analysis confirms the findings from the other population genetic analyses (Figure 3b). Krennkogel was again resolved as somewhat distinct. The Ochsenstein and Glitzalm subpopulations, however, do show quite some overlap in the DAPC plot, despite significant population genetic differentiation, as inferred by F_ST_.

**Figure 4:**
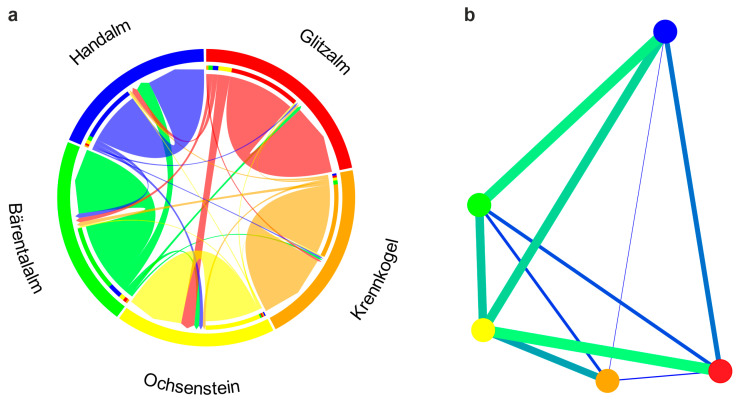
Population connectivity in *Salamandra atra* from the Koralpe, Austria. (**a**) Circus plot visualization of recent migration rates inferred in BayesAss. (**b**) Population network (EDENetwork with a percolation threshold of 0.1) based on estimates of population genetic differentiation (F_ST_). Population (nodes) are linked by edges that are weighted/colored in proportion to F_ST_ values.

Recent migration rates, as inferred in BayesAss, were very low for most pairwise comparisons (Figure 4a). Higher migration rates were only inferred from Handalm to Bärentalalm and Glitzalm to Ochsenstein. Thus, these high migration rates concerned population pairs that showed the lowest level of differentiation. These high rates are likely an artifact, as with low levels of differentiation BayesAss is known to perform poorly [60,73]. Indeed, at least in the case of migration inference from Handalm to Bärentalalm a smaller peak close to 0 becomes evident in the migration rate distribution (Figure S3). Network visualization based on F_ST_ revealed a network with two clusters at the automatic percolation threshold of 0.03 (Figure S4). Again, one cluster included only the Krennkogel, whereas the other cluster comprised the remaining four sampling areas. Network visualization at a manual threshold of 0.1 connected all sampling areas (Figure 4b) and thus provided a more complete and more intuitive picture about the population connectivity of *S. atra* in the Koralpe mountain range. At this threshold level, *S. atra* from Krennkogel were still very much isolated, but showed the highest connectivity with the Ochsenstein population.

Using the LD based method in NeEstimator2, contemporary N_e_ was estimated to be 245 (95% CI 132–968) for the whole study region. No significant excess heterozygosity was observed for any of the five sampling areas (one-tailed Wilcoxon tests, *p* > 0.05), suggesting that *S. atra* in the study region did not experience a drastic reduction of N_e_ in the recent past. On the other hand, a mode shift in the allele frequency distribution might suggest a recent bottleneck for the Bärentalalm population (Table 3). Sample size, however, was not particularly large for this population, such that the observed mode shift might be due to biased allele frequency estimates resulting from inadequate sampling.

## 4. Discussion

The Alpine salamander from the Koralpe represents a genetically distinct and geographically isolated population at the southeastern margin of the species’ distribution range. Our integrative analysis combining mitochondrial and microsatellite data reveals a high degree of genetic structuring among sampling sites and confirms the absence of shared haplotypes with other conspecific populations across Austria and the broader Alpine region. These findings align with previous work highlighting the species’ pronounced phylogeographic structure and limited dispersal capacity, traits that jointly underscore the evolutionary and conservation significance of peripheral populations such as those from the Koralpe.

The identification of two unique mitochondrial haplotypes that are confined to Alpine salamanders from the Koralpe is notable. While Alpine salamanders from this region clearly cluster within the nominate subspecies *S. a. atra*, the absence of haplotype sharing with other hitherto sequenced Austrian, Italian, or Slovenian populations suggests a history of prolonged isolation and restricted geneflow. These patterns are consistent with the broader phylogeographic framework of *S. atra*, where historical climatic fluctuations and the topographic complexity of the Alps have driven lineage diversification and the persistence of unique haplogroups in glacial microrefugia [19,21].

Our microsatellite analyses revealed strong population differentiation and a pronounced lack of connectivity among sampling localities on the Koralpe. Currently, the wind-exposed ridgelines between the sampled populations exhibit habitat conditions largely unfavorable to the Alpine salamander. For most parts, there are no spring outlets, hiding places, rocky structures, or deadwood present. In addition, the landscape is subject to intensive grazing. Despite some efforts between 2020 and 2022, no Alpine salamanders were found there (even though the spatial extent of the single populations is larger than covered by our sampling) [74], and therefore these areas between the sampled populations do not represent gaps in survey coverage, but rather areas with a lack or very low densities of Alpine salamander. Although there are no direct barriers between the sampled areas, the absence of stepping-stone habitats and overall unfavorable habitat conditions have the same isolating effect. Adult *S. atra* are largely philopatric and have home ranges of just a few hundred square meters [43,75,76], such that even short stretches of inhospitable habitat represent effective barriers to dispersal and, hence, geneflow. Indeed, overall levels of inferred recent geneflow were generally low among the Koralpe populations, with single larger geneflow estimates being most likely artifacts due to the generally low, but nonetheless significant, estimates of population genetic differentiation [60,73]. The geographic distances between our five sampled areas on the Koralpe ranged from less than one to a few kilometers. This high degree of structure over relatively short spatial scales—taking into account that the actual spatial distance between the distinct populations is smaller than evident from our sampling [74]—not just mirrors, but rather excels findings from other peripheral populations of *S. atra* in the Dinarides and Prealps [14,20,21], where genetic discontinuities occurring across distances of <10 km have been attributed to low dispersal capacity and strong habitat fidelity. Despite being largely unfavorable to the species as permanent habitat, migration and, hence, geneflow between populations appears to happen predominately over alpine meadows rather than spruce and/or mountain pine forests (Figure 1 and Figure 4b). Interestingly, although geographically closest to other subpopulations, the Krennkogel subpopulation is the most genetically differentiated. This may be due to an exposed mountain ridge separating it from nearby subpopulations, potentially acting as an effective barrier to gene flow. Alpine salamanders not only have a limited dispersal capacity, but also a low reproductive potential, with females giving birth to one or two offspring after a gestation period of two to four years, depending on the altitude [13,77,78,79]. This implies that it takes some time until populations become significantly differentiated from each other. Hence, the high level of population genetic differentiation at very small spatial scales in *S. atra* from the Koralpe likely does not reflect recent anthropogenic impact, but rather reflects longstanding isolation. Unfortunately, no mutation rates are available for microsatellites of this species (or any closely related species), precluding the estimation of reliable divergence times among the *S. atra* populations from the Koralpe mountain range.

With an estimated effective population size (N_e_) of 245 (95% CI: 132–986) across the entire study area, the whole *S. atra* population from the Koralpe is not particularly small, but also cannot be considered very large. Even though the five sampling areas are significantly differentiated, the absolute degree of population differentiation is rather low and should have only a minor impact on the N_e_ estimation, as the method is robust to a wide range of scenarios [67]. If there was an effect due to spatial structure, it would be an overestimation of the true N_e_ [68]. On the other hand, overlapping generations, which is very likely also the case for our *S. atra* samples—*S. atra* starts to reproduce at 3–4 years and can reach an age of about 15 years [80]—slightly underestimate the true N_e_ [81]. No severe recent genetic bottlenecks were detected; the only potential indication for a bottleneck comes from a mode shift in the allele frequency distribution in the Bärentalalm population. Generally, according to the 50/500 rule, it is assumed that an N_e_ > 50 is necessary to prevent short-term extinction due to inbreeding depression and the negative effects of genetic drift (e.g., random fixation of deleterious alleles), but an N_e_ > 500 is needed to maintain adaptive potential—that is, the ability to adapt to changing environmental conditions—over the long term [82,83]. There is some debate about the general applicability of this rule. In particular, some researchers suggest that the 50/500 rule is not sufficient and should be increased to 100/1000 [84]. Sample sizes for individual sub-populations were too small to reliably infer N_e_ (i.e., negative N_e_ estimates for three subpopulations, and 95% CI included infinity for all subpopulations), but it is safe to assume that N_e_ of the single subpopulations is smaller than for the whole Koralpe population. Consequently, isolation and habitat fragmentation still pose significant long-term risks. Small N_e_ values can compromise adaptive potential, increase susceptibility to stochastic events, and elevate the probability of inbreeding depression over time—especially under accelerating environmental change [7,8]. Although overall heterozygosity was moderately high compared to other *S. atra* populations [14,20,21], localized reductions in allelic richness at some sites highlight the need for continued monitoring.

Ecologically, the Koralpe population occupies a biogeographically significant area within the southeastern Alps, a region proposed as a glacial refugium for cold-adapted taxa during the Last Glacial Maximum [25,26]. The Alpine salamander is adapted to a cold and moist environment, with long reproductive cycles and low levels of activity and, hence, low dispersal capacity. However, these same ecological and life-history traits impose constraints on population expansion and recolonization potential, making the Koralpe population particularly vulnerable to future climate-induced habitat loss. High-resolution models predict substantial declines in suitable habitat for *S. atra* in the coming decades [85,86,87], particularly in the southeastern Alps, which will not only experience a dramatic temperature increase, but also a decrease in precipitation [88]. While on the short term the *S. atra* populations at the Koralpe might even benefit from increasing temperatures (not taking into account likely associated changes in the general habitat) by shorter gestation times and, hence, increased reproductive potential, the species will experience increasing pressure in the long term. Already, the species is inhabiting the higher parts of the Koralpe mountain range, with little room for further uphill movement with rising temperatures.

From a conservation genetics perspective, the Alpine salamanders from the Koralpe population meet key criteria for recognition as a discrete management unit (MU), and possibly as an evolutionarily significant unit (ESU), pending further genomic investigation. Following the framework proposed by [89], the combination of historical isolation, absence of geneflow, and unique genetic variants supports its consideration as a conservation priority. Such isolated populations contribute disproportionately to the species’ overall genetic and evolutionary diversity, and their loss would represent an irreversible erosion of *S. atra*’s phylogeographic legacy. Moreover, given the role of peripheral populations as potential sources of novel adaptations to changing environments [90], their protection may have implications beyond local biodiversity conservation. Future conservation strategies should prioritize the in situ protection of current *S. atra* populations on the Koralpe. Efforts should also aim to maintain or restore landscape connectivity where feasible, taking into account the species’ low dispersal capacity. Importantly, we advocate for continued genetic monitoring to detect early signs of genetic erosion or demographic instability.

Our study has limitations that merit future research investment. Although nine microsatellite markers provide valuable insights into neutral population genetic structure, they lack the resolution to detect selection-driven variation or fine-scale demographic shifts. Employing population genomic approaches would significantly enhance our ability to detect demographic patterns on a much finer scale. Longitudinal sampling across years or decades would reveal dynamic patterns in N_e_, subpopulation persistence, colonization events, or habitat-related demographic fluctuations. Finally, acquisition of functional genomic data—such as genes relevant to thermal tolerance or desiccation resistance—will be essential for interpreting ecological resilience under projected climate change scenarios.

## 5. Conclusions

The Alpine salamander population from the Koralpe exemplifies how historical biogeographic processes and contemporary ecological constraints interact to shape fine-scale genetic structures in montane amphibians. Its genetic distinctiveness, demographic independence, and peripheral location within a climate-sensitive region strongly argue for its inclusion in national and EU-level conservation frameworks. The preservation of such unique populations is not only essential for maintaining the evolutionary potential of *S. atra*, but also for sustaining the broader genetic integrity of Europe’s alpine biota in an era of rapid environmental change.

## Figures and Tables

**Table 1:** Microsatellite diversity in *Salamandra atra* populations from the Koralpe, Austria.

Population						Locus					Average (s.d.)
		SalE12	SalE6	SalE8	SST-C3	SST-E11	SST-G6	Sal23	SalE7	SalE14	
HA (N = 21)	N_A_	6	6	2	4	9	2	4	7	6	5.11 (2.32)
	H_O_	0.857	0.810	0.381	0.571	0.810	0.211	0.714	0.762	0.619	0.637 (0.218)
	H_E_	0.775	0.722	0.372	0.553	0.830	0.341	0.612	0.800	0.763	0.640 (0.184)
BA (N = 16)	N_A_	5	3	2	4	10	2	4	4	7	4.56 (2.56)
	H_O_	0.875	0.563	0.250	0.500	0.875	0.286	0.538	0.917	0.625	0.603 (0.247)
	H_E_	0.800	0.599	0.229	0.464	0.780	0.349	0.726	0.764	0.823	0.615 (0.219)
OS (N = 13)	N_A_	7	7	3	5	8	1	4	6	6	
	H_O_	0.769	0.9230	0.308	0.923	0.846	NA	0.692	0.615	0.769	0.731 (0.201)
	H_E_	0.828	0.766	0.283	0.757	0.840	NA	0.742	0.680	0.837	0.717 (0.184)
KK (N = 20)	N_A_	4	5	2	5	7	2	3	6	4	4.22 (1.72)
	H_O_	0.700	0.550	0.050	0.750	0.700	0.450	0.400	0.500	0.700	0.533 (0.221)
	H_E_	0.673	0.709	0.050	0.745	0.629	0.512	0.650	0.685	0.763	0.602 (0.220)
GA (N = 20)	N_A_	5	6	2	4	9	2	4	4	6	4.67 (2.18)
	H_O_	0.750	0.500	0.300	0.700	0.850	0.300	0.650	0.350	0.750	0.572 (0.214)
	H_E_	0.700	0.664	0.328	0.696	0.787	0.431	0.668	0.455	0.753	0.609 (0.162)
average	N_A_	5.4	5.4	2.2	4.4	8.6	1.8	3.8	5.4	5.8	
total	N_A_	7	8	3	6	15	2	5	7	10	

N, number of samples; N_A_, number of alleles per locus; H_O_, observed heterozygosity; H_E_, expected heterozygosity; no significant deviations from Hard–Weinberg expectations were observed after correction for multiple testing [47]. HA, Handalm; BA, Bärentalalm; OS, Ochsenstein; KK, Krennkogel; GA, Glitzalm.

**Table 2:** Pairwise population differentiation (F_ST_) between the five populations of *Salamandra atra* from the Koralpe, Austria, based on nine microsatellites.

Population	HA	BA	OS	KK
**BA**	0.0236 **			
**OS**	0.0318 **	0.0362 **		
**KK**	0.0947 ***	0.0770 ***	0.0467 **	
**GA**	0.0618 ***	0.0618 ***	0.0185 *	0.0900 ***

Significance levels, *p* < 0.05, <0.01 and <0.001, after correction for multiple tests [47], are indicated as *, ** and ***, respectively. HA, Handalm; BA, Bärentalalm; OS, Ochsenstein; KK, Krennkogel; GA, Glitzalm.

**Table 3:** *p*-values for test for heterozygote excess based on the TPM (95% SMM; variance in the size of nonstepwise mutations = 12) and inferred mode shift in the allele frequency distribution for *Salamandra atra* populations from the Koralpe, Austria.

Population	TPM	Mode Shift
HA	0.213	No
BA	0.326	Yes
OS	0.629	No
KK	0.367	No
GA	0.590	No

HA, Handalm; BA, Bärentalalm; OS, Ochsenstein; KK, Krennkogel; GA, Glitzalm.

## Data Availability

DNA sequence data generated in this study are available from GenBank under the accession numbers PX021649-PX021676 (cytb) and PX021677-PX021704 (CR); information on previously published DNA sequence data used in the present manuscript is available in Table S2. The microsatellite data used for the population genetic analyses are available in Table S3.

## Supplementary Materials

The following supporting information can be downloaded at: https://www.mdpi.com/article/10.3390/biology14101428/s1, Table S1: Sample list, with information on sampling locality GenBank accession numbers and whether microsatellite data could be generated; Table S2: Information on DNA sequence data of *S. atra* used in the current study; Table S3: Microsatellite data for *S. atra* from the Koralpe mountain range; Table S4: Information on primer combinations, primer concentration and annealing temperature for PCR amplification of microsatellites; Figure S1: ML tree of *S. atra* haplotypes; Figure S2: Statistical support for K clusters; Figure S3: Pairwise migration rates as inferred by BayesAss; Figure S4: Population network (EDENetwork with an automatic percolation threshold of 0.03) based on estimates of population genetic differentiation (F_ST_).
