# Supplementary material for: Unique and Under Pressure: Conservation Genetics of an Isolated Alpine Salamander Population
Source: Biology (Basel). 2025 Oct 17;14(10):1428. doi: 10.3390/biology14101428 (PMC12562145; doi:10.3390/biology14101428)
Supplement: Supplementary file 1 [file biology-14-01428-s001.zip › Figure S1.pdf]

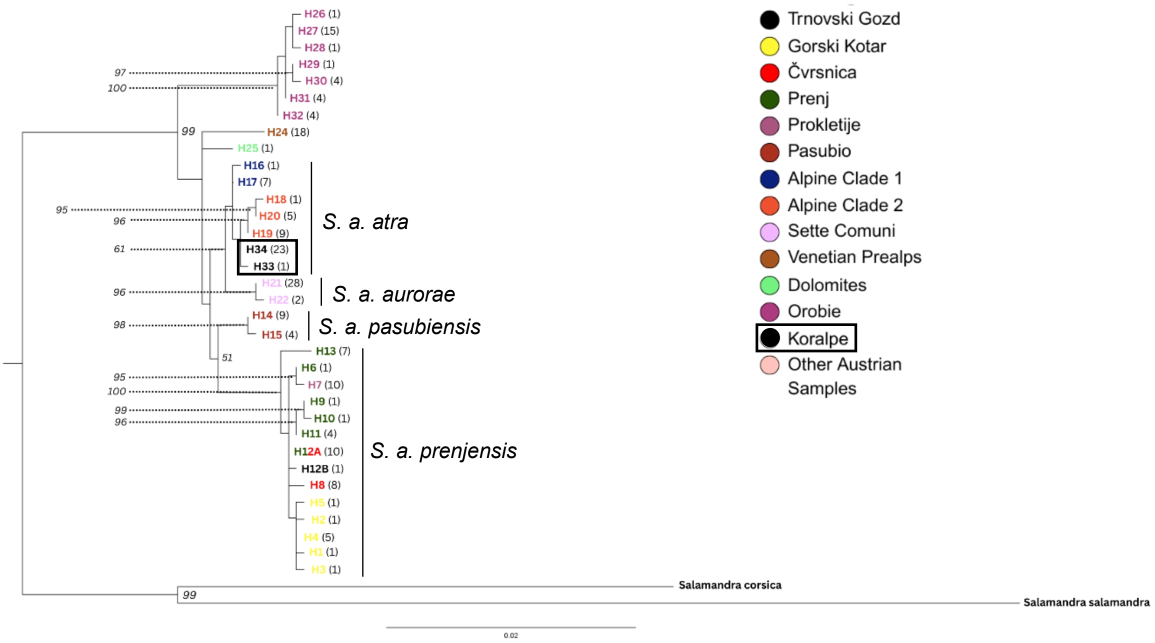

Figure S1. Maximum likelihood tree of the *Salamandra atra* haplotypes (concatenated cytb plus CR alignment). Only bootstrap values >50 are shown. The numbers in parentheses next to the haplotype IDs refer to the number of samples-
