# Supplementary material for: Unique and Under Pressure: Conservation Genetics of an Isolated Alpine Salamander Population
Source: Biology (Basel). 2025 Oct 17;14(10):1428. doi: 10.3390/biology14101428 (PMC12562145; doi:10.3390/biology14101428)
Supplement: Supplementary file 1 [file biology-14-01428-s001.zip › Figure S2.pdf]

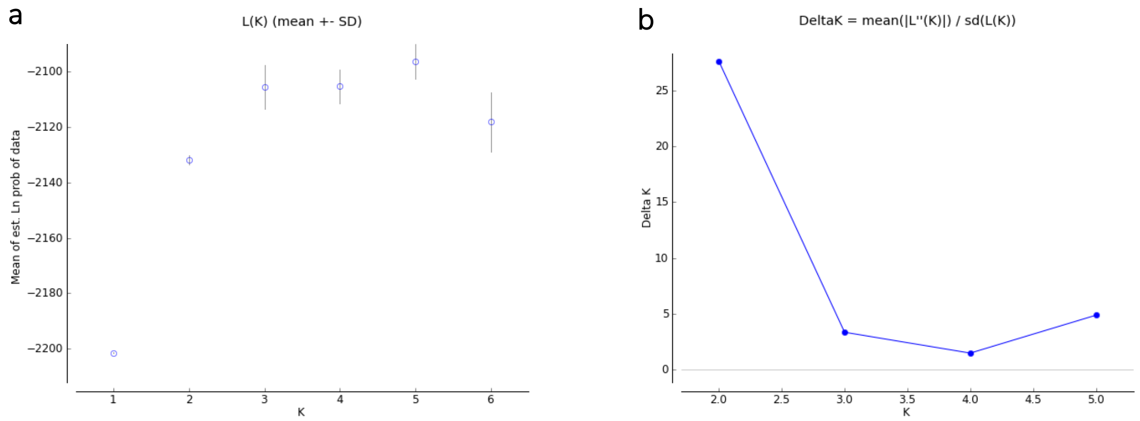

Figure S2. Statistical support for K clusters. Mean likelihood [L(K) ± SD] over 10 runs assuming K clusters (a) and DeltaK, where the modal value of the distribution is considered to represent the highest level of structuring (b).
