# Supplementary material for: Unique and Under Pressure: Conservation Genetics of an Isolated Alpine Salamander Population
Source: Biology (Basel). 2025 Oct 17;14(10):1428. doi: 10.3390/biology14101428 (PMC12562145; doi:10.3390/biology14101428)
Supplement: Supplementary file 1 [file biology-14-01428-s001.zip › Figure S4.pdf]

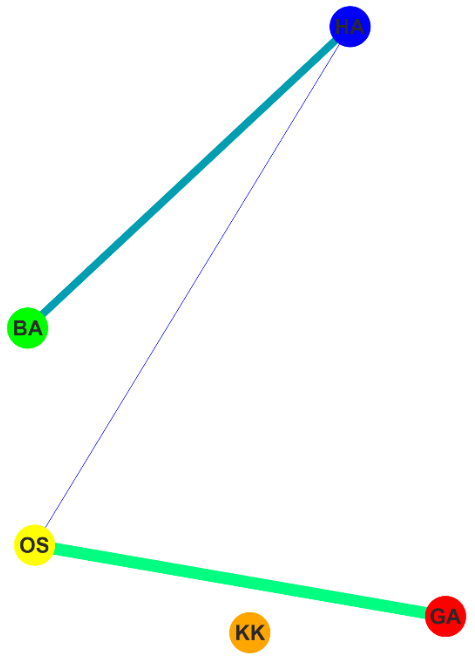

Figure S4. Population network (EDENetwork with with an automatic percolation threshold of 0.03) for *Salamandra atra* populations from the Koralpe, Austria, based on estimates of population genetic differentiation (F_{ST}). Population (nodes) are linked by edges that are weighted/colored in proportion to F_{ST}s. HA, Handalm; BA, Bärentalalm; OS, Ochsenstein; KK, Krennkogel; GA, Glitzalm
