# Supplementary material for: Unique and Under Pressure: Conservation Genetics of an Isolated Alpine Salamander Population
Source: Biology (Basel). 2025 Oct 17;14(10):1428. doi: 10.3390/biology14101428 (PMC12562145; doi:10.3390/biology14101428)
Supplement: Supplementary file 1 [file biology-14-01428-s001.zip › Table S4.pdf]

Table S4. Information on primer combinations, primer concentration and annealing temperature for PCR amplification of microsatellites.

single-/multiplex	locus	primer name	primer [µM]	annealing temperature (°C)
P3A	SS-C3	SST-C3_F_6FAM	0.17	59
		SST-C3-R		
	SST-E11	SST-E11_ATTO550	0.33	
		SST-E11_R		
	SST-G6	SST-G6_F_ATTO565	0.33	
		SST-G6_R		
P3B	SaIE6	SaIE6_F_ATTO550	0.27	60
		SaIE6_R		
	SaIE8	SaIE8_F_6FAM	0.17	
		SaIE8_R		
	SaIE12	SaIE12_F_ATTO565	0.33	
		SaIE12_R		
P2C	SaIE7	SaIE7_F_ATTO565	0.17	54
		SaIE7_R		
	SaI23	SaI23_F_FAM	0.17	
		SaIE23_R		
P1D	SaIE14	SaIE14_F_6FAM	0.42	64
		SaIE14_R		
